# Antifouling Effects of Superhydrophobic Coating on Sessile Marine Invertebrates

**DOI:** 10.3390/ijerph19137973

**Published:** 2022-06-29

**Authors:** Seongjun Bae, Ye Ju Lee, Min Kyung Kim, Yeongwon Kwak, Chang-Ho Choi, Dong Gun Kim

**Affiliations:** 1 Department of Ecology and Conservation, Marine Biodiversity Institute of Korea, Seocheon 33662, Korea; silverto@mabik.re.kr; 2 Department of Ocean Environmental Sciences, College of Natural Science, Chungnam National University, Daejeon 34134, Korea; 3 Institute of Environmental Ecology, Sahmyook University, Seoul 01795, Korea; helloyj1985@gmail.com; 4 Department of Bio & Environment Technology, Seoul Women’s University, Seoul 01797, Korea; min4190@naver.com; 5 Department of Chemical Engineering, Gyeongsang National University, Jinju 52828, Korea; hyjk755@gnu.ac.kr (Y.K.); ch_choi@gnu.ac.kr (C.-H.C.); 6 Smith College of Liberal Arts, Sahmyook University, Seoul 01795, Korea

**Keywords:** marine biofouling, fabric substrate, superhydrophobic, biomimetic antifouling, *Ascidiella aspersa*, *Bugula neritina*

## Abstract

Biofouling is a significant problem in the aquaculture and marine shipping industries; thus, various antifouling methods have been developed to prevent the resultant economic losses. In the present study, the superhydrophobic surface of a lotus leaf was bio-mimicked to achieve antifouling. Specifically, fabric substrates with and without superhydrophobic coatings on the surface were installed on the Tongyeong yacht in December 2020 (group A) and April 2021 (group B), and the coverage of the attached invertebrates was recorded every month until August 2021. The coverage of solitary ascidians (*Ascidiella aspersa* and *Ciona robusta*) and branching bryozoans (*Bugula neritina*) was lower on the coated substrates than on the non-coated ones, and coating or non-coating was significantly correlated with the extent of coverage. Superhydrophobic substrates with a low surface energy and micro–nano dual structure may be unsuitable for the attachment of larvae. Therefore, superhydrophobic coating is a more effective and simpler method of antifouling for certain taxa than other antifouling strategies. However, the antifouling effect of the superhydrophobic substrate in group A reduced after 5 months from the first installation; thus, the durability of the antifouling coating should be further improved, and solving this problem remains a major task, necessitating further research.

## 1. Introduction

In aquatic environments, submerged solid surfaces are prone to the colonization of sessile benthic invertebrates, such as macroalgae, sponges, polychaetes, barnacles, mollusks, bryozoans, and ascidians, which release planktonic larvae or spores that attach to new substrates [1,2]. This phenomenon is called marine biofouling. Typically, biofouling causes many problems in the aquaculture and marine shipping industries. Specifically, these invertebrates cause pollution by attaching to the infrastructure of aquaculture farms [3], or increase drag, fuel consumption, and operating costs by attaching to cargo ships [4]. Therefore, to prevent such economic losses, many antifouling strategies—including physical, chemical, and biological methods—have been developed [5]. A common antifouling method involves the inclusion of heavy metals and co-biocides in the paint matrix [6]. Tributyltin (TBT), a self-polishing copolymer, was proven to be the most effective coating material; however, its commercial use was banned in 2008 due to severe negative environmental effects [7]. Another common antifouling method involves the coating of materials containing copper or organotin compounds, which can effectively prevent the settlement of benthic invertebrates by leaching biocides [8]. However, similar to the case of TBT, demand to restrict the use of copper-based antifouling paints is growing at present due to their persistence, bioaccumulation, and other adverse environmental impacts [9,10,11,12].

In this context, more environmentally friendly technologies are being explored. For instance, silica mesoporous nanocapsules (SiNCs) contain a biocide inside them, lowering their associated environmental risks [10]. Photodynamic antifouling technology is still used only in laboratories, and has no practical applications, but it is one of the representative environmentally friendly antifouling systems [13,14]. Silicon-based fouling-release coatings prevent the formation of strong bonds on surfaces with low surface energy, modulus, and microroughness [15]. Additionally, in recent years, biomimetic antifouling strategies have been widely studied. One of the strategies is to mimic the “lotus effect”—that is, the superhydrophobic property of the surface of lotus leaves. The superhydrophobic surface of lotus leaves has garnered much attention, and to mimic this property, a micro–nano dual structure and low surface energy are integrated [5]. Such superhydrophobic surfaces are resistant to stains, proteins, bacteria, proteins, and marine organisms [16,17], offering many advantages and enabling wide applications [18] due to their excellent self-cleaning, anti-freeze [19], and anti-corrosion [20,21] properties. Pechook et al. [22,23] created superhydrophobic surfaces via high-temperature deposition of paraffin wax and fluorine wax on various surfaces—such as copper, glass, and silicon—to form nano-shaped layers. Zheng et al. [24] used a natural lotus leaf as a template and copied it using a molding method to create a lotus leaf polyurethane surface. Chen et al. [25] synthesized superhydrophobic FOTS-TiO_2_ particles via solvothermal synthesis with a surface morphology similar to that of a lotus leaf, which can be used to manufacture superhydrophobic coatings on various substrates.

To this end, the present study aimed to confirm the antifouling effect of an environmentally friendly superhydrophobic coating using a relatively simple and large-scale spray-coating method. Briefly, coating was achieved via two-step spray deposition. The first layer was composed of a TiO_2_–epoxy resin nanocomposite, which improved the robustness of the coating. In the second layer, fluorocarbon–silane-modified SiO_2_ nanoparticles (FC–silane SiO_2_ NPs) were deposited. Functionalized silica NPs were used to construct the micro–nano dual structure. In a laboratory-based pre-study, the superhydrophobic surface with a contact angle of 0.6° remained durable at 3.6° after exposure to water and saltwater for up to 13 days under agitation at 500 rpm [5]. To test whether the superhydrophobic coating produced an antifouling effect on sessile benthic invertebrates even in a marine environment with various other factors, fabric substrates with this coating were installed at a depth of 5 m in a harbor. Furthermore, to confirm the duration of the antifouling effect, monthly scuba surveys were conducted from November 2020 to August 2021. Overall, our goal was to determine whether the superhydrophobic coating produced antifouling effects against marine organisms, and for how long the antifouling effect of the coating lasted in the marine environment.

## 2. Materials and Methods

### 2.1. Experimental Design

**Figure 1:**
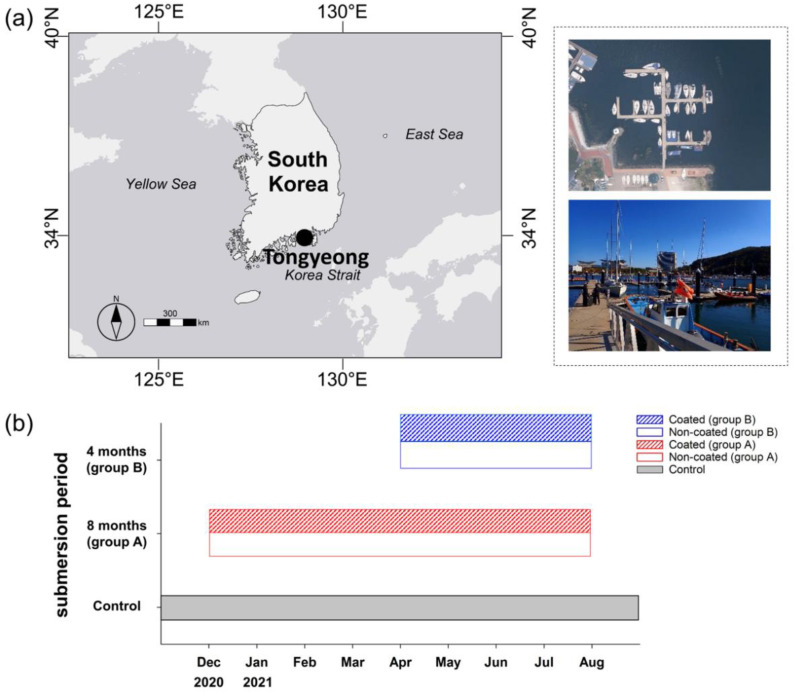
Map showing the location of the survey site, including satellite and overview photographs (**a**). Graph showing the submersion period of the substrate in each group (**b**).

The present study was conducted at the Marine Sports Center (34°49′38.80″ N, 128°26′4.22″ E) in the city of Tongyeong (Figure 1a) in Gyeongsangnam-do in the southern part of the Korean Peninsula. Study sites were located in places where yachts and ships are loaded in the harbor, and various artificial structures—such as port walls and floating docks—exist. The control group was an acrylic substrate (300 × 300 mm), and was installed on three columns at the yacht yard in April 2017. Three columns were selected as replicates, and fabric substrates (300 × 300 mm) with and without superhydrophobic coatings on the surface were installed at a depth of 3–4 m in December 2020 (group A) and April 2021 (group B). The contact angle of the superhydrophobic surface was 0.6°. After installing the fabric substrate in December 2020, eight scuba surveys were conducted every month from January 2021, and the coverage of the attached invertebrates was recorded until August 2021. Biological factors (i.e., species richness and coverage) were recorded in the survey log, and photographs were obtained using a camera (ZV-1, Sony, Japan) to estimate the changes in recruitment and species composition over time. In addition, the depth of the substrate was estimated at each point using a dive computer (Descent™ Mk1, Garmin Ltd., Olathe, KS, USA) during the field survey.

In addition, to observe the durability of the superhydrophobic coating, an experiment was performed by dividing the submersion period of the substrate into 8 (group A) and 4 (group B) months. After immersion for 4 months following the installation of group A, group B was installed, and the antifouling effects of group A and group B were observed. Therefore, the substrates in group A were submerged for 8 months, from December 2020 to August 2021, while those in group B were submerged for 4 months, from April to August 2021 (Figure 1b).

### 2.2. Fabrication and Characterization of the Superhydrophobic Coating

Titanium (IV) oxide (powder, 99.8%), silica (SiO_2_) (fumed), and trichloro(1H,1H,2H,2H-perfluorooctyl)silane (PFOCTS, 97%) were purchased from Sigma-Aldrich. Acetone (99.5%) and 1-propanol (99.0%) were purchased from SAMCHUN Chemical Co., Ltd. Toluene (99.5%) was purchased from JUNSEI. Epoxy resin and hardener were purchased from Dasol Scientific Co. Ltd. The main components of the epoxy resin and hardener were 2,2-bis (4′-glycidiyloxyphenyl) propane (diglycidyl ether of bisphenol A) and trimethylolpropane poly(oxypropylene)triamine, respectively.

To fabricate the superhydrophobic surface, SiO_2_ NPs were functionalized with fluorocarbon, as described previously [26]. Briefly, 2 g of SiO_2_ NPs was dispersed in 41 mL of 40:1 toluene/PFOCTS mixture. The dispersion was refluxed for 3 h to produce FC–silane SiO_2_ NPs. Following reflux, the FC–silane SiO_2_ NPs were washed with ethanol several times and then dried at 70 °C in an oven. To prepare the first coating suspension, TiO_2_ NPs (8.3 g) and epoxy resin (40 g) were dispersed in 40 mL of acetone solvent for 15 min under magnetic stirring. Then, the hardener (20 g) was added to the dispersion, followed by stirring for an additional 5 min. To formulate the second coating solution, FC–silane SiO_2_ NPs (0.5 g) were dispersed in 50 mL of 1-propanol via sonication for 30 min. Then, 90 mL of the first coating suspension was sprayed on the fabric substrate using an airbrush, followed by curing in the oven at 70 °C for 6 h. Finally, 45 mL of the second coating solution was sprayed onto the cured first coating layer and subjected to a second curing in an oven at 70 °C for at least 1 h.

### 2.3. Data Analyses

Species were identified in the field. Relevant encyclopedias and literature were consulted to confirm the identity of small individuals or to validate taxa that were difficult to identify [27,28,29,30,31] based on photographic data. Each taxon was analyzed by grouping at the phylum or class level for statistical analysis. Bryozoans and ascidians were divided into two groups according to the morphotype attached to the substrate. Bryozoans were categorized as branching or encrusting according to their structure and disarticulation/fragmentation style. Branching organisms grow vertically, and the entire colony is flexible. In contrast, encrusting organisms form single- or multilayered hard calcareous structures [32,33,34]. Furthermore, ascidians were categorized morphologically and ecologically as solitary or colonial. Solitary ascidians are independent individuals and reproduce by sexual reproduction, whereas colonial ascidians form colonies and reproduce both sexually and asexually via budding and strobilation (Table S1) [35,36].

Average values of species richness and coverage (coverage percent) from three replicates were used in all data analyses, and rare species (i.e., those with a frequency < 5%) were excluded from the analyses. Coverage was calculated using the grid cell counting method of photoQuad, version 1.4 [37], based on photographic data in the laboratory. Permutational multivariate analysis of variance (PERMANOVA) was run on a triangular similarity matrix derived from the Bray–Curtis dissimilarity index of square-root-transformed data. PERMANOVA was performed as a two-way analysis to verify the significance of month and coating as factors. To obtain the graphical ordination of benthic invertebrates, non-metric multidimensional scaling (nMDS) was used, and the data were relativized for species coverage to the same portion [38]. We used multiple response permutation procedure (MRPP)—a nonparametric protocol—to test the null hypothesis of no difference between the groups in the data matrix and determine the significance of the differences between coated and non-coated substrates. Similarity percentage (SIMPER) analysis was used to compare the coverage of species in groups A and B for identifying the species that contributed the most to the differences between coated and non-coated substrates. Student’s *t*-test was used to compare the coverage of benthic invertebrates between coated and non-coated substrates. Statistical significance was set at *p* < 0.05. PERMANOVA was conducted with the PERMANOVA+ add-on package in PRIMER-e, version 6 (www.primer-e.com) [39,40]. nMDS and MRPP analyses were conducted using PC-ORD, version 6, Gleneden Beach, OR, USA [41]. Student’s *t*-test was performed using R, version 4.0.2. Vienna, Austria [42], and SIMPER was performed using the vegan package in R [43].

## 3. Results

Throughout the entire study period, including groups A and B, 23 species of marine benthic invertebrates belonging to 19 families in 14 orders, 8 classes, and 8 phyla were observed. After 4 rare species were excluded from the analyses, the number of species was 12 in group A, 9 in group B, and 19 in the control group (Table S2). In PERMANOVA of three groups (group A, B, and total), significant temporal differences were noted in group A (*p* = 0.0001), but there was no significant difference between the coated and non-coated substrates (*p* = 0.0721). Meanwhile, in group B, both temporal differences and differences between the coated and non-coated substrates were significant (*p* = 0.0002; Table 1).

**Figure 2:**
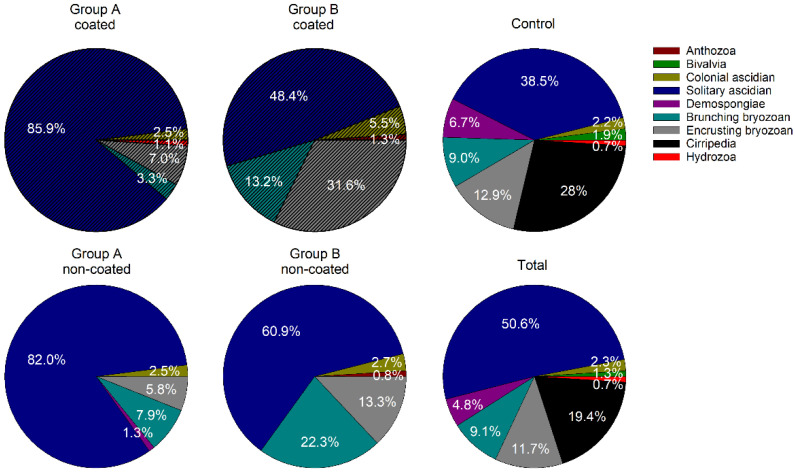
Pie charts showing the accumulated coverage of each taxon in each sample group measured within the survey period. Each wedge indicates the relative overall coverage of each taxon, and only shows cumulative coverage greater than 0.5%.

Solitary ascidians, including *A. aspersa* and *C. robusta*, were the dominant taxa with the highest mean coverage, followed by the branching bryozoan *B. neritina*. In particular, the coverage was relatively high in July (summer). In July, the mean coverage of *A. aspersa* and *B. neritina* on the non-coated substrate in group A was 57.6% and 16.5%, respectively. Among all taxa, Anthozoa, Cirripedia, and Hydrozoa did not exceed 5% mean coverage during the study period in groups A and B, nor in the control group. During the initial colonization period (1–4 months), the mean coverage in groups A and B was markedly lower than that in the control group. After May, as the total biomass increased, the mean coverage in groups A and B as well as the control group showed a similar trend. However, the branching bryozoans in groups A and B showed a different trend from those in the control group, as an exception. In July and August, the mean coverage was approximately 10% higher on the non-coated substrate in groups A and B (Figure 2 and Figure S1).

**Figure 3:**
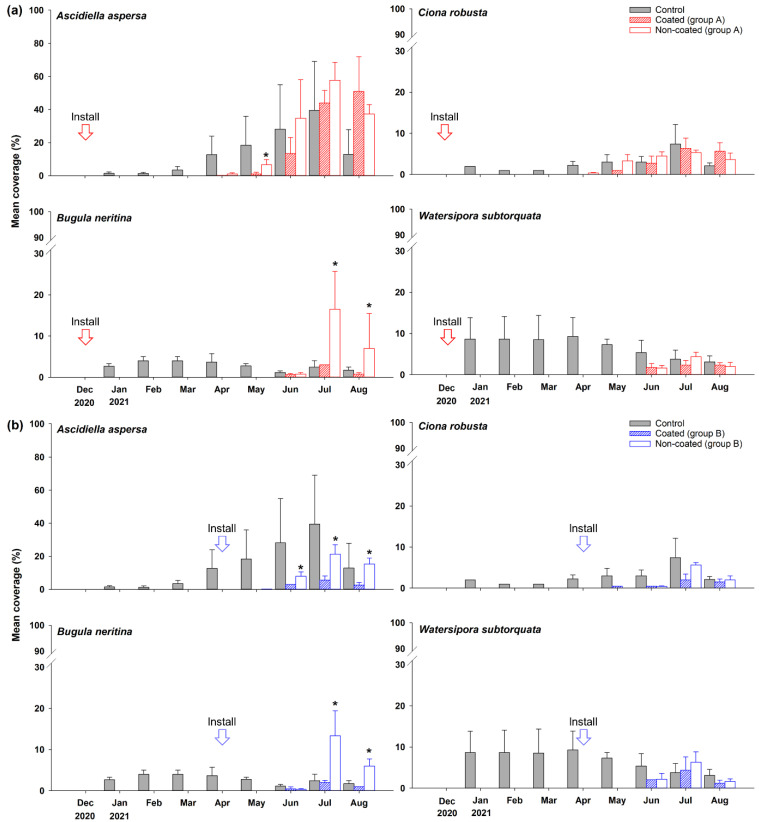
Mean coverage of four species with large contributions to differences between coated and non-coated substrates in groups A (**a**) and B (**b**). Asterisks indicate significant differences.

We then analyzed the contribution of species to the significant differences between the coated and non-coated substrates using SIMPER analysis. *A. aspersa* made the greatest contribution in both groups A and B, producing the greatest effect on differences in coverage between the coated and non-coated substrates. Next, the species making the second-greatest contribution were *C. robusta* in group A and *B. neritina* in group B. In particular, the top three dominant species in group A were colonial ascidians and branching bryozoans, with high mean coverage during summer. The species making the third-greatest contribution in group B was *Watersipora subtorquata*—an encrusting bryozoan. Therefore, the top four species that contributed the most to the differences between coated and non-coated substrates in groups A and B were *A. aspersa*, *C. robusta*, *B. neritina*, and *W. subtorquata* (Table 2). In group A, *A. aspersa* and *C. robusta* showed significant differences between the coated and non-coated substrates (*p* < 0.05), with a higher coverage on non-coated than on coated substrates in May. After May, there were no significant differences among the solitary ascidians in group A (*p* > 0.05); however, in group B, the coverage of *A. aspersa* was higher on non-coated than on coated substrates during June–August (*p* < 0.05). Similarly, significant differences were noted in the coverage of *C. robusta* in group B during July (*p* < 0.05). In both groups A and B, the coverage of *B. neritina* was higher on non-coated substrates in July and August (*p* < 0.05). Conversely, in both groups A and B, the coverage of *W. subtorquata* did not significantly differ between the coated and non-coated substrates during the study period (*p* > 0.05; Figure 3).

**Figure 4:**
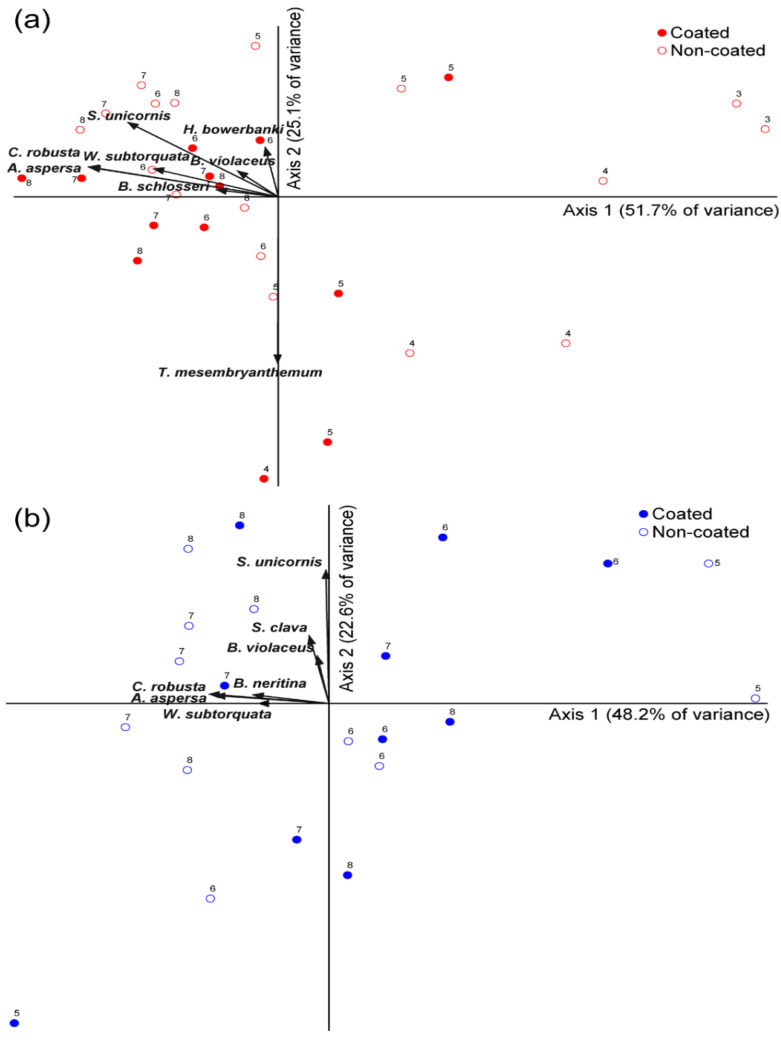
Results of nMDS analysis showing differences in sessile invertebrate communities between coated (close) and non-coated (open) substrates during the study period. Taxa present in over 5% of the samples are included. Biplot shows the linear relationships between nMDS ordinates and species coverage. The red and blue dots indicate groups A (**a**) and B (**b**), respectively. Each number indicates a time point.

Furthermore, our nMDS results were consistent with the SIMPER results. In group A, there were differences in colonization in terms of temporal changes, although the plot was not divided based on the presence or absence of a coating. However, in group B, the plots for the coated and non-coated substrates were divided by axis 1. As such, the coverage of *A. aspersa* and *C. robusta* was particularly affected during June–August (Figure 4). MRPP analysis comparing the coverage between coated and non-coated substrates revealed a significant difference in group B (*p* = 0.01623), but not in group A (*p* = 0.12232) or the overall sample (*p* = 0.12522; Table 3).

## 4. Discussion

In the present study, we applied a robust superhydrophobic coating on a fabric substrate for effective management of biofouling, and examined its effects on the recruitment of benthic invertebrates. South Korea, which is in East Asia, has four seasons, and temporal variations in environmental factors affect species community compositions. Therefore, we analyzed the effects of different factors by dividing the temporal variations and coatings into two experimental groups. In PERMANOVA analysis, significant differences were noted in all three study groups due to temporal changes, and both species coverage and community structure were affected by environmental factors. Meanwhile, there was a significant difference in the temporal factor in all groups, and the environmental change according to the season was considered to have an effect. The superhydrophobic coating showed a significant difference only in group B and the whole group, but not in group A. This was attributed to specific taxa of group B showing significant differences between coated and non-coated, while in the case of group A, it was probably because the increase in the coverage of the taxa was less affected by the coating after summer (Table 1).

Among all taxa, only solitary ascidians and branching bryozoans showed lower coverage on the substrate due to the effect of the superhydrophobic coating, while there were no differences in the coverage of other taxa. This pattern may be attributed to differences in attachment characteristics across taxa or species. The shapes of bryozoan, ascidian, and barnacle larvae attached to substrates are different, and their length varies in the range of 120–500 μm [44]. In addition, the preference of different substrates for settlement differs between species. For instance, the bryozoan *Membranipora membranacea* prefers high altitudes to avoid competition for space [45], while the solitary ascidian *Styela canopus* prefers cryptic habitats [46]. Furthermore, the roughness of the substrate’s surface affects its antifouling effect. As such, Scardino et al. [47] reported that the nanostructure of the superhydrophobic coating substrate was effective in antifouling against algal cells, algal spores, bryozoans, and barnacles. In addition, barnacle and mussel larvae recognize settlement substrates by positively responding to chemical signals released by the former or present colonies of the same species [48,49]. Consequently, the antifouling effect of a coating material may be restricted to specific taxa, because the physical and chemical preferences of larvae differ between species.

In a previous study on the attachment behavior of *Ciona* larvae—one of the solitary ascidians—a hydrophilic surface negatively affected attachment, while a hydrophobic surface was preferred for attachment [50,51]. However, in the present study, the coverage of solitary ascidians on the superhydrophobic-coated surface was lower than that on the non-coated surface. Likewise, contrary to previous reports that branching bryozoans prefer hydrophobic surfaces [50,52], our results showed their lower coverage on the superhydrophobic-coated substrate (Figure 3 and Figure S1). The superhydrophobic coating likely produced a negative physical and chemical effect on the attachment and colonization of ascidian and bryozoan larvae because of its micro–nano dual structure and low surface energy [5]. Thus, our findings corroborate previous speculations that superhydrophobicity exerts potent antifouling effects through reducing the adhesive strength by providing a smaller adhesion area and lowering the effective concentration of antifouling compounds [19,53,54]. In a previous study that confirmed the difference in coverage of invertebrates with an antifouling system with ZnO particle coating, the coverage of the entire taxa with the ZnO coating was statistically significantly lower than that of the control group [55]. Although the ZnO particle coating was more effective in antifouling against benthic invertebrates than the superhydrophobic coating, it is considered that the application potential of the superhydrophobic coating is greater due to the biocides issued from the metal coating. In addition, it is more eco-friendly than the lotus leaf effect antifouling using wax or polyurethane [22,23,24], and because our coating method uses a spray application, it can be deposited on the substrate more easily than the antifouling using FOTS-TiO_2_ particles [25].

In our nMDS analysis, only points during June–August in group B could be differentiated based on the presence or absence of a coating along axis 1. This supports our observation of significant differences during June–August in group B due to changes in the coverage of sessile benthic invertebrates (Figure 4). In SIMPER analysis, the top three species with large contributions in groups A and B were solitary ascidians and bryozoans; thus, the coverage of these two taxa contributed to the differences between the coated and non-coated substrates (Table 3). Our nMDS and SIMPER results were consistent in that the coverage of these two taxa differed on superhydrophobic-coated and non-coated substrates (Figure S1). In particular, the coverage of *A. aspersa*, *C. robusta*, and *B. neritina* was significantly different in the *t*-test. The coverage of *A. aspersa* differed significantly between the coated and non-coated substrates in group A only during May, while significant differences after May were observed only in group B. Similarly, the coverage of *C. robusta* differed only during May in group A, and only during July after May in group B. During June–August, group A was already exposed to the natural environment for 6 months after the first installation of the substrates, whereas group B was exposed only for 2–4 months during June–August after the first installation in April (Figure 3). Therefore, the performance of the superhydrophobic coating likely deteriorated after approximately 5 months. The superhydrophobic coating presents a micro–nano dual structure. Being highly delicate, this structure can be easily destroyed by physical impact. In particular, it is considered that the performance of the superhydrophobic coating continues to deteriorate in a marine environment where various complex environmental variables exist, such as temperature, pressure, and chemical corrosion [56]. Compared to the copper-based antifouling paint, which lasts for up to two years [57], the durability of the superhydrophobic coating is insufficient. However, research on various means of durability improvement is being conducted for an eco-friendly antifouling system. In previous studies, a polymer film [58] or a complex coating composed of perfluorinated SiO_2_ NPs and polydimethylsiloxane (PDMS) elastomer [59] was used to compensate for this shortcoming. Another strategy is to graft a diblock copolymer of poly(glycidyl methacrylate) and poly(2, 2, 2-trifluoroethyl methacrylate) with silane [60]—both studies conducted indoors. In field studies, Feng et al. [61] reported that the contact angle of the copper-based superhydrophobic coating material exposed to air for 10 months was lower than 8°, while Sakhuja et al. [62] reported that the antifouling efficiency decreased by only 0.3% when the hydrophilic nanostructured glass substrate was exposed to the outdoors for 3 months. In the present experiment, the first layer was composed of a TiO_2_–epoxy resin nanocomposite, and FC–silane SiO_2_ NPs were subsequently deposited to improve durability through double deposition. However, compared to that during pre-study in the laboratory, the environment in the field is complex, and it is difficult to perfectly simulate the actual study in the real environment. For this reason, the antifouling durability is considered to be limited, and the problem of short-term antifouling durability remains a major challenge to large-scale applications. Therefore, additional research and development are needed to solve the durability problem.

## 5. Conclusions

The present study analyzed differences in the coverage of marine benthic invertebrates between superhydrophobic-coated and non-coated substrates. The coverage of *Ascidiella aspersa*, *Ciona robusta*, and *Bugula neritina* was lower on the superhydrophobic-coated substrates than on the non-coated substrates. Superhydrophobic surfaces provide a smaller adhesion area, ultimately reducing the adhesion strength of larvae and leading to effective antifouling. We also considered that the antifouling effect differed between taxa, as larval preferences for physical and chemical substrates differ between taxa. After May 2021, the coverage of *A. aspersa* and *C. robusta* differed significantly only in group B. Thus, the performance of the superhydrophobic coating likely deteriorated after approximately 5 months. Being highly delicate, the micro–nano dual structure of the superhydrophobic coating can be destroyed by physical impact. Therefore, based on the results of a pre-study in the laboratory, we sought to increase durability using a resin nanocomposite. However, since the marine environment is complex and there are many additional variables, durable antifouling solutions remain a major challenge.

## Figures and Tables

**Table 1:** PERMANOVA results showing differences in month and coating.

Group	Variable	Mean Coverage (%)				
		df	SS	MS	Pseudo-F	*p* (perm)
A	Month	5	51783	10,357	14.0300	**0.0001**
	Coat	1	1601.3	1601.3	2.1693	0.0721
	Month × coat	4	10,150	2537.5	3.4376	**0.0004**
	Residual	19	14,025	738.16		
B	Month	3	17,513	5837.7	4.6959	**0.0002**
	Coat	1	8152.7	8152.7	6.5581	**0.0002**
	Month × coat	3	11,921	3973.6	3.1964	**0.0005**
	Residual	13	16,161	1243.1		
Total	Month	5	52,205	10,441	5.5328	**0.0001**
	Coat	1	6217.5	6217.5	3.2948	**0.0058**
	Month × coat	4	10,588	2647	1.4027	0.1014
	Residual	40	75,483	1887.1		

Bold values denote significant results.

**Table 2:** Results of SIMPER analysis showing species contributions to differences between coated and non-coated substrates in groups A and B.

Group	Species	Average Group Abundances		Average Contribution	SD of Contribution	Average to SD Ratio
		Coated	Non-Coated			
A	*Ascidiella aspersa*	26.376	26.311	0.431	0.261	1.651
	*Ciona robusta*	3.692	3.300	0.065	0.068	0.955
	*Bugula neritina*	0.730	2.852	0.033	0.055	0.607
	*Tubularia mesembryanthemum*	0.392	0.158	0.029	0.108	0.272
	*Watersipora cucullata*	1.615	1.411	0.027	0.029	0.939
	*Schizoporella unicornis*	0.853	0.682	0.024	0.064	0.376
B	*Ascidiella aspersa*	2.800	12.200	0.341	0.167	2.035
	*Bugula neritina*	1.040	4.727	0.122	0.101	1.202
	*Watersipora subtorquata*	1.850	2.772	0.098	0.093	1.056
	*Ciona robusta*	0.800	1.709	0.059	0.082	0.727
	*Schizoporella unicornis*	0.630	0.427	0.038	0.067	0.567
	*Botrylloides violaceus*	0.050	0.645	0.027	0.054	0.499

**Table 3:** Results of MRPP analysis comparing the coverage between coated and non-coated substrates in three groups.

Comparison Factor	Group	Test Statistic (T)	Chance-Corrected within-Group Agreement (A)	*p*-Value
Coated × non-coated	A	−1.13686	0.02043	0.12522
	B	−2.61232	0.07362	**0.01623**
	Total	−1.13916	0.01152	0.12232

Bold values denote significant results.

## Data Availability

Not applicable.

## Supplementary Materials

The following supporting information can be downloaded at: https://www.mdpi.com/article/10.3390/ijerph19137973/s1, Figure S1: Mean coverage of each taxon observed during the study period; Table S1: Species lists for the ascidian and bryozoan groups; Figure S2. A photograph of the colonization process of benthic invertebrates attached to the substrate. After substrate installation, one month and seven months in group A (a), and one month and four months in group B (b); Table S2. List of sessile marine invertebrates observed at survey sites during the study period. Asterisks indicate rare species (i.e., those occurring at a frequency < 5%) excluded from the analysis. Open circles indicate species observed in each group.
