# Supplementary material for: Antifouling Effects of Superhydrophobic Coating on Sessile Marine Invertebrates
Source: Int J Environ Res Public Health. 2022 Jun 29;19(13):7973. doi: 10.3390/ijerph19137973 (PMC9265409; doi:10.3390/ijerph19137973)
Supplement: Supplementary file 1 [file ijerph-19-07973-s001.zip › ijerph-1764389-supplementary.pdf]

Supplementary Information

Antifouling Effects of Superhydrophobic Coating on Sessile Marine Invertebrates

Seongjun Bae [1,2,†], Ye Ju Lee [3,†], Min Kyung Kim [4], Yeongwon Kwak [5], Chang-Ho Choi [5], and Dong Gun Kim [6,*]

[1]Department of Ecology and Conservation, Marine Biodiversity Institute of Korea, Seocheon 33662, Republic of Korea

[2]Department of Ocean Environmental Sciences, College of Natural Science, Chungnam National University, Daejeon 34134, Republic of Korea

[3]Institute of Environmental Ecology, Sahmyook University, Seoul 01795, Republic of Korea

[4]Department of Bio & Environment Technology, Seoul Women's University, Seoul 01797, Republic of Korea

[5]Department of Chemical Engineering, Gyeongsang National University, Jinju 52828, Republic of Korea

[6]Smith Liberal Arts College, Sahmyook University, Seoul 01795, Republic of Korea

[†]Seongjun Bae and Ye Ju Lee contributed equally to this work.

[*]Correspondence to: Dong Gun Kim (ecology@syu.ac.kr)

Supplementary Information includes
Figure S1
Tables S1 and S2

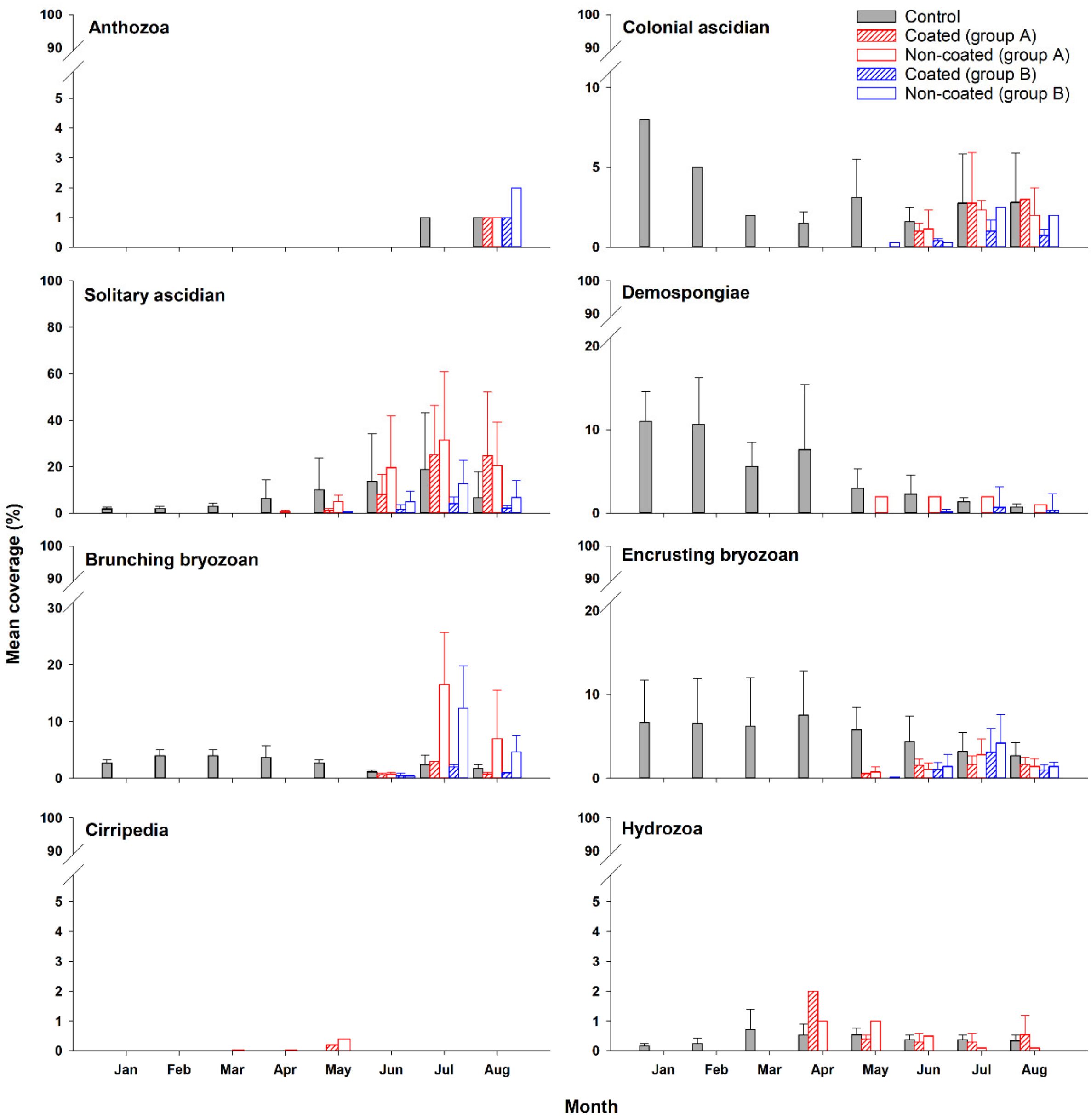

Figure S1. Mean coverage of each taxon observed during the study period.

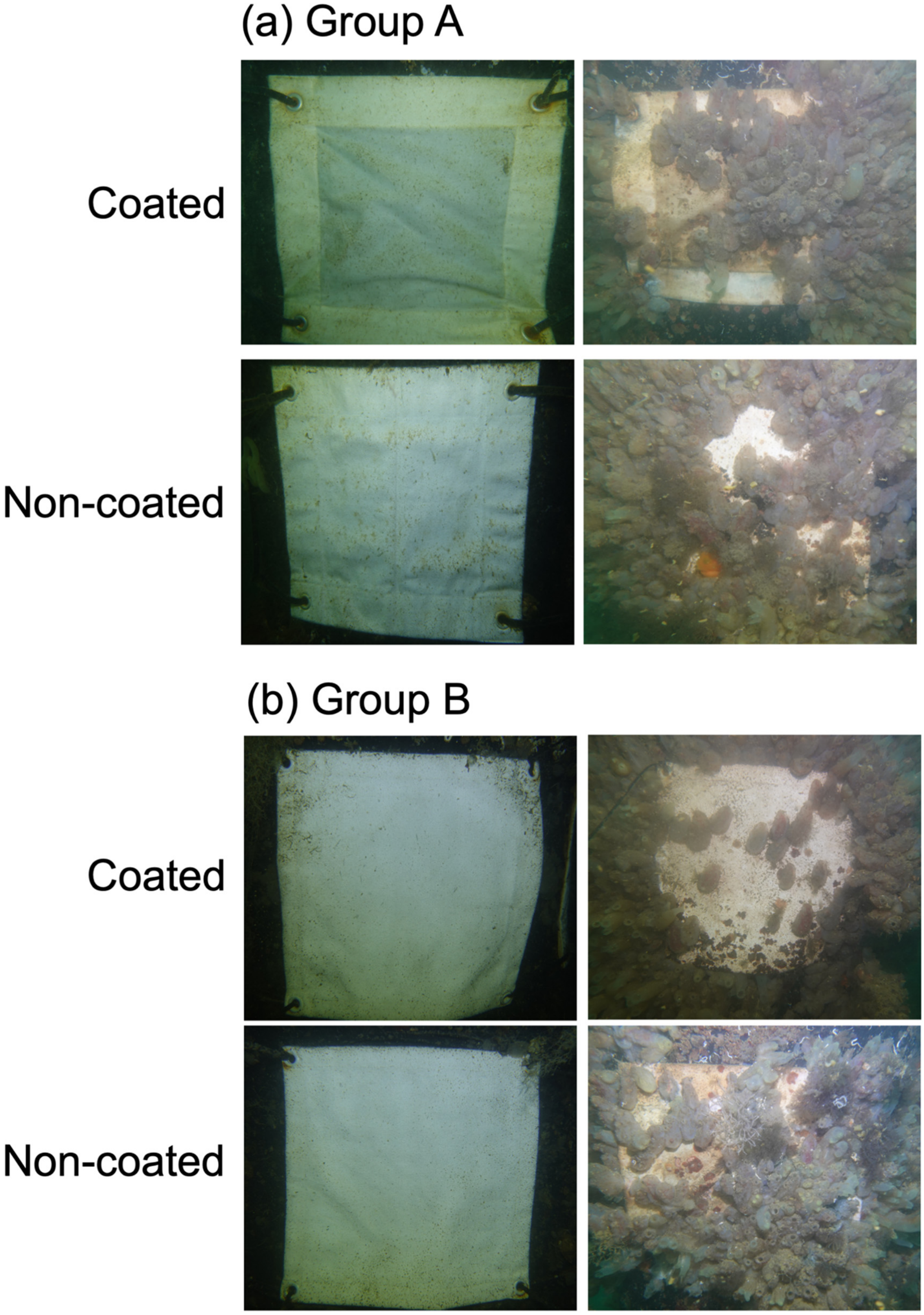

Figure S2. A photograph of the colonization process of benthic invertebrates attached to the substrate. After substrate installation, one month and seven months in group A (a), and one month and four months in group B (b).

Table S1. Species lists for the ascidian and bryozoan groups.

Taxon group	Ascidian group		Bryozoan group	
	Solitary	Colonial	Branching	Encrusting
Species list	*Ascidiella aspersa*<br>*Ciona robusta*<br>*Ciona savignyi*<br>*Halocynthia roretzi*<br>*Styela clava*	*Botrylloides violaceus*<br>*Didemnum vexillum*<br>*Botryllus schlosseri*	*Bugula neritina*<br>*Tricellaria occidentalis*	*Schizoporella unicornis*<br>*Watersipora subtorquata*

Table S2. List of sessile marine invertebrates observed at survey sites during the study period. Asterisks indicate rare species (i.e., those occurring at a frequency of < 5 %) excluded from the analysis. Open circles indicate species observed in each group.

Taxa \ Group	Control	A	B
Phylum Porifera			
Class Demospongiae			
Order Suberitida			
Family Halichondriidae			
Halichondria (*Halichondria*) *bowerbanki*	○	○	
Phylum Cnidaria			
Class Hydrozoa			
Order Anthoathecata			
Family Tubulariidae			
Tubularia mesembryanthemum	○	○	
Order Leptothecata			
Family Campanulariidae			
Obelia dichotoma	○		
Class Anthozoa			
Order Actiniaria			
Family Actiniidae			
Anthopleura anjunae	○	○	○
Phylum Mollusca			
Class Bivalvia			
Order Mytilida			
Family Mytilidae			
Mytilus galloprovincialis	○		
Order Ostreida			
Family Ostreidae			
Magallana gigas	○		
Order Pectinida			
Family Anomiidae			
*Pododesmus macroschisma**	○		
Phylum Arthropoda			
Class Hexanauplia			
Order Sessilia			
Family Balanidae			
Balanus trigonus	○	○	
Fistulobalanus albicostatus	○		
Phylum Cnidaria			
Class Anthozoa			
Order Actiniaria			
Family Actiniidae			
*Anthopleura anjunae**	○	○	○
Phylum Bryozoa			
Class Gymnolaemata			
Order Cheilostomata			
Family Schizoporellidae			
Schizoporella unicornis	○	○	○

Table S2. Continued

Taxa \ Group	Control	A	B
Phylum Bryozoa			
Class Gymnolaemata			
Order Cheilostomatida			
Family Bugulidae			
Bugula neritina	○	○	○
Family Candidae			
*Tricellaria occidentalis**	○	○	○
Family Watersiporidae			
Watersipora subtorquata	○	○	○
Phylum Chordata			
Class Ascidiacea			
Order Aplousobranchia			
Family Didemnidae			
Didemnum vexillum	○		
Order Phlebobranchia			
Family Ascidiidae			
Ascidiella aspersa	○	○	○
Family Cionidae			
Ciona robusta	○	○	○
Ciona savignyi	○		
Order Stolidobranchia			
Family Botryllidae			
Botrylloides violaceus	○	○	○
Family Pyuridae			
Halocynthia roretzi	○		
Family Styelidae			
Botryllus schlosseri	○	○	○
Styela clava	○	○	○
*Styela plicata**		○	
